# Genome of *Leptospira borgpetersenii* strain 4E, a highly virulent isolate obtained from *Mus musculus* in southern Brazil

**DOI:** 10.1590/0074-02760170111

**Published:** 2018-02

**Authors:** Marcus Redü Eslabão, Frederico Schmitt Kremer, Rommel Thiago Juca Ramos, Artur Luiz da Costa da Silva, Vasco Ariston de Carvalho Azevedo, Luciano da Silva Pinto, Éverton Fagonde da Silva, Odir Antônio Dellagostin

**Affiliations:** 1 Universidade Federal de Pelotas, Núcleo de Biotecnologia, Capão do Leão, RS, Brasil; 2 Universidade Federal do Pará, Belém, PA, Brasil; 3 Universidade Federal de Minas Gerais, Belo Horizonte, MG, Brasil; 4 Universidade Federal de Pelotas, Faculdade de Veterinária, Capão do Leão, RS, Brasil

**Keywords:** bioinformatics, genomics, leptospirosis, neglected disease, whole genome shotgun sequencing

## Abstract

A previous study by our group reported the isolation and characterisation of *Leptospira borgpetersenii* serogroup Ballum strain 4E. This strain is of particular interest because it is highly virulent in the hamster model. In this study, we performed whole-genome shotgun genome sequencing of the strain using the SOLiD sequencing platform. By assembling and analysing the new genome, we were able to identify novel features that have been previously overlooked in genome annotations of other strains belonging to the same species.

The *Leptospira* genus consists of 23 species of bacteria (Boonsilp et al. 2013, Bourhy et al. 2014), of which at least nine are naturally pathogenic, five are opportunists (“intermediary pathogenic”), and the remaining are saprophytes (non-pathogenic). *L. interrogans* is the most commonly reported cause of leptospirosis, which is an infection caused by pathogenic leptospiras; however, other species, such as *L. borgpetersenii*, *L. kierschneri*, and *L. santarosai*, are also associated with leptospirosis and are responsible for many infections and deaths both in humans and animals (Guerra 2009). Leptospirosis is a worldwide distributed zoonotic disease that has reemerged as a public health problem in many countries in recent years, especially in countries located in the tropics (Guerra 2013).

*L. borgpetersenii* serovar Ballum strain 4E was isolated from the suburban area of Pelotas, a city located in southern Brazil, from mice (*Mus musculus*) (da Silva et al. 2010). Previous studies have demonstrated that this strain has a LD50 (lethal dose for 50% of the population) of ~5.18 leptospires in a hamster model. As such, it is more lethal and virulent than are other standard model strains such as *L. interrogans* serovar Copenhageni strain Fiocruz L1-130 (LD50 = ~80 leptospires) (Diniz et al. 2011). The characterisation of highly virulent strains may provide useful data that can potentially extend our knowledge and understanding of the pathogenesis of these bacteria and lead to the development of new vaccines. Further, it may generate insights that are useful for epidemiological surveillance. In the present study, we performed a whole-genome shotgun analysis of the *L. borgpetersenii* serovar Ballum strain 4E to develop a more comprehensive characterisation of this isolate.

Bacterial culture and DNA extraction were performed in accordance with previously described methods (Kremer et al. 2016b). Whole-genome shotgun sequencing was performed using the ABI SOLiD v. 4 sequencing platform with a 50 base-pair (bp) single-end library.

Raw reads in colour-space FASTA format (csFASTA) were pre-processed using SAET (https://www.thermofisher.com/) and converted into FASTQ format using our in-house Python script cs2q (http://labbioinfo.ufpel.edu.br/cs2q).

Two assembly approaches were evaluated for the *L. borgpetersenii* strain 4E genome: *de novo* assembly and reference-guided assembly. *De novo* assembly was performed using Velvet, with different parameters of k-mer length, expected coverage and coverage cutoff, and the assembly metrics were accessed using QUAST (Gurevich et al. 2013). Reference-guided assembly was performed by mapping the reads to the genome of *L. borgpetersenii* serovar Ballum strain 56604 (GenBank: CP012029.1, CP012030.1) using SMALT (www.sanger.ac.uk/science/tools/smalt-0 *)*. The resulting SAM file was then converted to BAM format and sorted using Samtools before a consensus sequence was extracted using Samtools, BCFtools, VCFutils.pl (Li et al. 2009) and GATk (McKenna et al. 2010). Genome annotation was performed using Genix (Kremer et al. 2016a) and manually reviewed and curated using Artemis (Rutherford et al. 2000).

A variant calling analysis using Samtools, BCFtools, and VCFutils.pl that was based on the BAM file generated from the aligned reads was performed to identify single nucleotide polymorphisms (SNPs) and insertions and deletions (INDELs). The effect of each variant was inferred based on the annotation of *L. borgpetersenii* serovar Ballum strain 56604 using Snpeff (Reumers 2004).

The reference-guided assembly covered > 99.99% of the reference sequence, with a mean coverage of ~ 400x. A lack of coverage was identified in five assembly gaps, which were associated with mobile elements, such as transposons, that can change their positions in the genome and usually result in gaps in reference-guided assemblies or collapses in a single contig in *de novo* assembly from short reads, even when they are present in multiple copies. The *de novo* assemblies generated by Velvet were highly fragmented, with more than 5,000 contigs and a very low N50 (53), thus making it inappropriate for any downstream analysis.

An overview of the features identified in the genome of *L. borgpetersenii* serovar Ballum strain 4E is shown in Table I. We identified a total of 3469 coding DNA sequences (CDSs), 37 transfer-RNAs (tRNAs), 4 ribosomal RNAs (rRNAs), one transfer-messenger RNA (tmRNA) and five riboswitch *loci*. Although the proteincoding genes found were almost the same as those identified in the genome of the 566604 strain, by using our annotation pipeline, we were able to identify new non-coding features that were overlooked in the reference annotation: a tmRNA gene and riboswitches. TmRNAs act as tRNAs and contain a small open reading frame (ORF) in their structure that encodes a peptide responsible for many regulation processes, including targeting proteins for degradation (Hayes & Keiler 2010). Riboswitches are non-coding motifs that are present in the untranslated regions (UTRs) of some messenger RNAs (mRNAs) that act as cis-regulatory elements and bind specific metabolites to inhibit the gene expression. Riboswitches are typically found in genes associated with vitamin metabolism, e.g., cobalamin (Garst et al. 2011, Serganov & Nudler 2013). Previous studies have demonstrated that riboswitch-regulated cobalamin (B12) autotrophy is a virulence factor in the *Leptospira* genus (Fouts et al. 2016). Therefore, a deeper annotation of the non-coding features may provide a better description of the resulting transcriptome.

**TABLE I:** Features identified in the draft genome of the *Leptospira borgpetersenii* serovar Ballum strain 4E during the annotation

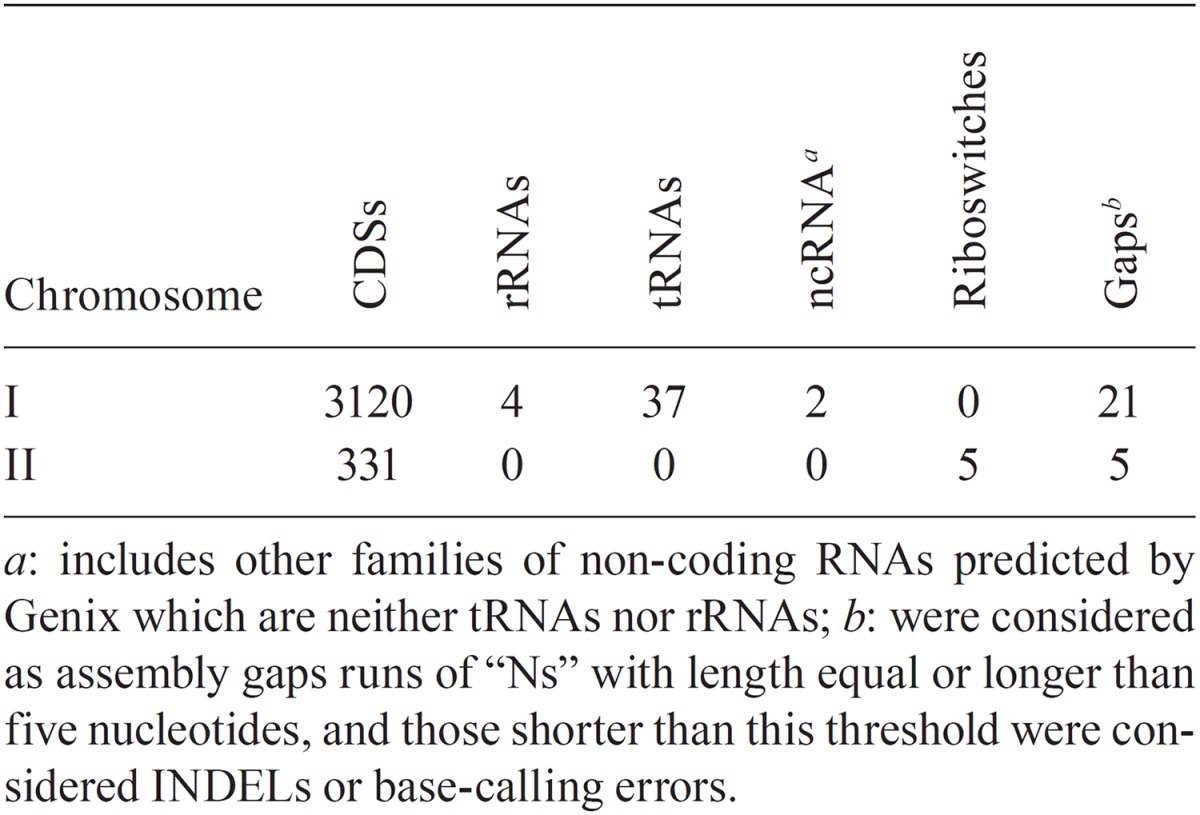

The genes that presented missense mutations in the variant calling analysis are displayed in Table II, and their locations in the genome of *L. borgpetersenii* strain 4E are illustrated in Figure. A total 41 genes were predicted as being affected by missense mutations in the variant calling analysis, although 33 of them had only one mutation. One of the genes, LB4E_3373, which encodes a protein from the PF07598 family, presented 27 missense SNPs compared with the genome of the strain 56604. The orthologous genes from the PF07598 family have already been associated with adaptation to the host in *L. interrogans* and regulation of gene expression during the life cycle and infection (Lehmann et al. 2013).

**TABLE II:** Genes containing missense mutations identified in the genome of *Leptospira borgpetersenii* strain 4E based on the variant calling analysis using the genome of *L. borgpetersenii* strain 56604 as reference

Locus tag		SNPs	Product
Strain 56604	Strain 4E		
LBBP_04290	LB4E_3373	27	PF07598 family protein^a^
LBBP_02267	LB4E_1801	10	Hypothetical protein
LBBP_04295	LB4E_3378	5	Integrase core domain protein
LBBP_02266	LB4E_1800	5	M23 family peptidase^a^
LBBP_03954	-	3	Hypothetical protein
LBBP_02437	LB4E_1928	3	Hypothetical protein
LBBP_01389	LB4E_1117	3	PPE protein^a^
LBBP_04424	-	2	Hypothetical protein
LBBP_04423	LB4E_3488	1	Transcriptional regulator, Fis family
LBBP_04394	LB4E_3464	1	Putative EF-P lysine aminoacylase GenX
LBBP_04178	LB4E_3280	1	Transposase
LBBP_04103	LB4E_3222	1	AraC family transcriptional regulator
LBBP_03775	-	1	Hypothetical protein
LBBP_03455	LB4E_2709	1	PF07600 family protein
LBBP_03226	LB4E_2530	1	Flagellin domain protein^a^
LBBP_02875	LB4E_2269	1	Hypothetical protein
LBBP_02823	LB4E_2227	1	Transposase
LBBP_02742	LB4E_2163	1	Dolichyl-phosphate-mannose-protein mannosyltransferase
LBBP_02514	LB4E_1991	1	Stage II sporulation protein E
LBBP_02460	LB4E_1947	1	Hypothetical protein
LBBP_02259	LB4E_1792	1	DNA-directed RNA polymerase subunit beta
LBBP_01965	LB4E_1576	1	Ribosomal RNA small subunit methyltransferase H
LBBP_01593	LB4E_1288	1	1-aminocyclopropane-1-carboxylate deaminase
LBBP_01564	LB4E_1267	1	Tyrosine recombinase XerD
LBBP_01436	LB4E_1154	1	Oma87-like Outer membrane protein
LBBP_01392	LB4E_1120	1	Hypothetical protein
LBBP_01368	LB4E_1098	1	Hypothetical protein
LBBP_01318	-	1	Hypothetical protein
LBBP_01157	LB4E_1157	1	DNA repair protein RecN
LBBP_01063	LB4E_0848	1	tRNA nucleotidyltransferase/poly(A) polymerase family protein
LBBP_00977	LB4E_0776	1	Uncharacterized protein
LBBP_00916	LB4E_0716	1	Flagellar motor switch protein FliN
LBBP_00894	LB4E_0702	1	Transketolase, pyridine binding domain protein
LBBP_00821	LB4E_0643	1	Putative coproporphyrinogen dehydrogenase
LBBP_00739	LB4E_0580	1	Transposase
LBBP_00738	-	1	Hypothetical protein
LBBP_00468	-	1	Hypothetical protein
LBBP_00433	LB4E_0356	1	Hypothetical protein
LBBP_00376	LB4E_0309	1	Histidine kinase of a two-component regulator system
LBBP_00318	LB4E_0266	1	RND transporter, Hydrophobe/Amphiphile Efflux-1 (HAE1)/Heavy Metal Efflux (HME) family, permease protein
LBBP_00116	LB4E_0102	1	NUDIX hydrolase

a potentially related to pathogenesis.

**Figure:**
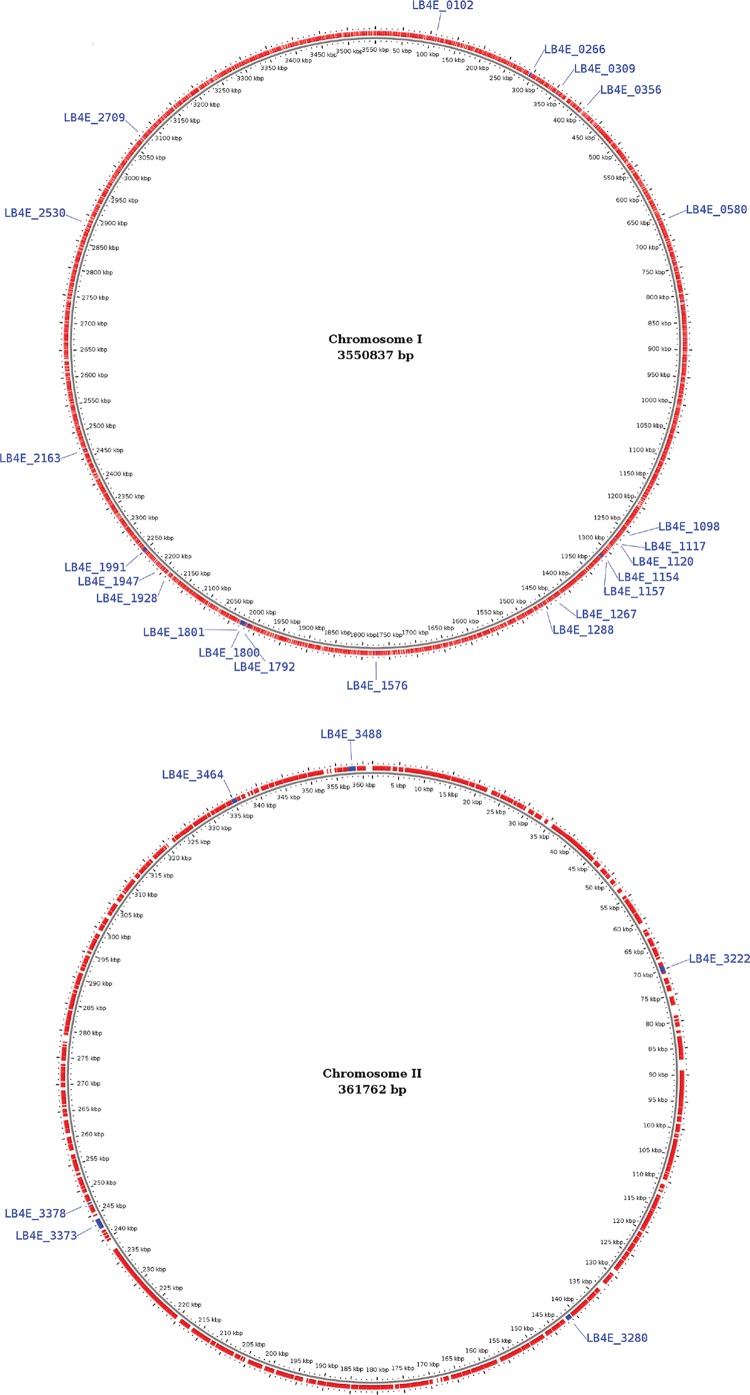
Map of the two chromosomes of *Leptospira borgpetersenii* strain 4E. Genes identified as mutated (non-synonymous mutations) based on comparison with *L. borgptersenii* strain 56604 are indicated in blue, and non-mutated genes are indicated in red.

Another highly mutated gene, LB4E_1801, contains 10 single-nucleotide polymorphisms, but its function remains unclear, and no BLAST hit in Uniprot (Apweiler et al. 2004) could allow a deeper annotation or provide any clue regarding its molecular function. We also identified five mutations in a gene that encodes an M23 peptidase (LB4E_1800), which has already been associated with fibronectin binding in *Leptospira* and other closely related genera, such as *Treponema*, and may contribute to the pathogenesis process.

Although *de novo* assembly is usually preferred for microbial organisms, it is associated with many drawbacks in obtaining a finished genome (Miller et al. 2010). Therefore, reference-guided assembly, based on an already-finished genome, may be a more reasonable approach to assembly when a closely related reference is available. In our case, both the 4E and 56604 strains belonged to the same species and serovar, so there was no requirement for a *de novo* assembly in this case. In fact, the SOLiD sequencing platform offers a high-throughput platform, short read length (50 bp) and high accuracy (Liu et al. 2012); as such, it is more suitable for re-sequencing/reference-guided assembly than *de novo* assembly.

The SOLiD sequencing process requires two hybridisation reactions to identify each base, so the probability of an erroneous identification or an artificial insertion / deletion tends to be much smaller compared with other platforms, such as Illumina and IonTorrent. In fact, in cases of sequencing artefacts, the decoding process of the colour-space data (csFASTA) to nucleotide-space format (FASTA) (based on nucleotide transitions) would generate an apparently random sequence after the erroneous base position, which probability would not align to the reference genome in the read mapping process (during a variant calling study) or be used in the assembly of a contig (in a *de novo* assembly). The reliability of this platform has already been demonstrated by previous studies, such as the benchmarking study performed by Ratan et al. (2013), which compared the accuracy of three different NGS platforms (ABI SOLiD, Illumina HiSeq and Roche 454 FLX) in the identification of SNPs in a human sample. In this case, the number of SNPs identified by SOLiD that were validated by mass-spectrometry was higher that what was observed in the other platforms. Therefore, although SOLiD is not a first option for microbial genomics, for which benchtop platforms are usually preferred, it may still be a valuable tool when aiming for a more accurate identification of mutations.

Finally, a *de novo* assembly using SOLiD data resulted in a more fragmented draft genome than other sequencing technologies because the short read length implies that there are many difficulties for the assembly algorithms due to the occurrence of repeated regions along the genome that may be collapsed by the *de Bruijn* graphs (Alkan et al. 2010); as such, this method would not be appropriate in this case.

In the context of *Leptospira* research, genomic data from highly virulent strains might provide useful information for the development of new vaccines and diagnostic methods and improve the understanding of bacterial pathogenesis and pathogen-host interactions. The presence of a high number of mutations in a gene that encodes a protein from the PF07598 family, which has already been suggested to be related to its pathogenesis in previous studies, may be one of the reasons for the greater virulence observed in this strain, although further studies are necessary to validate this relationship. Additionally, the availability of genomic characterisation from this strain might be useful for future epidemiological surveillance studies in southern Brazil.

*Nucleotide sequence accession number* - The complete genome of *L. borgpetersenii* strain 4E is available at GenBank under the accession codes CP015814.2 (chromosome I) and CP015815.2 (chromosome II). The raw reads from this sequencing project in are available at the NCBI Short Read Archive under accession code SRR5266483.

## References

[B1] Alkan C, Sajjadian S, Eichler EE (2010). Limitations of next-generation genome sequence assembly. Nat Methods.

[B2] Apweiler R, Bairoch A, Wu CH, Barker WC, Boeckmann B, O'Donovan C (2004). UniProt: the universal protein knowledgebase. Nucleic Acids Res.

[B3] Boonsilp S, Thaipadungpanit J, Amornchai P, Wuthiekanun V, Bailey MS, Holden MTG (2013). A single multilocus sequence typing (MLST) scheme for seven pathogenic *Leptospira* species. PLoS Negl Trop Dis.

[B4] Bourhy P, Collet L, Brisse S, Picardeau M (2014). *Leptospira mayottensis* sp. nov., a pathogenic species of the genus *Leptospira* isolated from humans. Int J Syst Evol Microbiol.

[B5] da Silva EF, Félix SR, Cerqueira GM, Fagundes MQ, Grassmann AA (2010). Preliminary characterization of *Mus musculus*-derived pathogenic strains of *Leptospira borgpetersenii* serogroup Ballum in a hamster model. Am J Trop Med Hyg.

[B6] Diniz JA, Félix SR, Bonel-Raposo J, Seixas ACP, Vasconcellos FA, Grassmann AA (2011). Highly virulent *Leptospira borgpetersenii* strain characterized in the hamster model. Am J Trop Med Hyg.

[B7] Fouts DE, Matthias MA, Adhikarla H, Adler B, Amorim-Santos L, Berg DE (2016). What makes a bacterial species pathogenic?: comparative genomic analysis of the genus *Leptospira*. PLoS Negl Trop Dis.

[B8] Garst AD, Edwards AL, Batey RT (2011). Riboswitches: structures and mechanisms. Cold Spring Harb Perspect Biol.

[B9] Guerra MA (2009). Leptospirosis. J Am Vet Med Assoc.

[B10] Guerra MA (2013). Leptospirosis: public health perspectives. Biologicals.

[B11] Gurevich A, Saveliev V, Vyahhi N, Tesler G (2013). QUAST: quality assessment tool for genome assemblies. Bioinformatics.

[B12] Hayes CS, Keiler KC (2010). Beyond ribosome rescue: tmRNA and co-translational processes. FEBS Lett.

[B13] Kremer FS, Eslabão MR, Dellagostin OA, Pinto LS (2016a). Genix: a new online automated pipeline for bacterial genome annotation. FEMS Microbiol Lett.

[B14] Kremer FS, Eslabão MR, Jorge S, Oliveira NR, Labonde J, Santos MNP (2016b). Draft genome of the *Leptospira interrogans* strains, Acegua, RCA, Prea, and Capivara, obtained from wildlife maintenance hosts and infected domestic animals. Mem Inst Oswaldo Cruz.

[B15] Lehmann JS, Fouts DE, Haft DH, Cannella AP, Ricaldi JN, Brinkac L (2013). Pathogenomic inference of virulence-associated genes in *Leptospira interrogans*. PLoS Negl Trop Dis.

[B16] Li H, Handsaker B, Wysoker A, Fennell T, Ruan J, Homer N (2009). The sequence alignment/map format and SAMtools. Bioinformatics.

[B17] Liu L, Li Y, Li S, Hu N, He Y, Pong R (2012). Comparison of next-generation sequencing systems. J Biomed Biotechnol.

[B18] McKenna A, Hanna M, Banks E, Sivachenko A, Cibulskis K, Kernytsky A (2010). The Genome Analysis Toolkit: a MapReduce framework for analyzing next-generation DNA sequencing data. Genome Res.

[B19] Miller JR, Koren S, Sutton G (2010). Assembly algorithms for next-generation sequencing data. Genomics.

[B20] Ratan A, Miller W, Guillory J, Stinson J, Seshagiri S, Schuster S (2013). Comparison of sequencing platforms for single nucleotide variants in a human sample. PLoS One.

[B21] Reumers J (2004). SNPeffect: a database mapping molecular phenotypic effects of human non-synonymous coding SNPs. Nucleic Acids Res.

[B22] Rutherford K, Parkhill J, Crook J, Horsnell T, Rice P, Rajandream M-A (2000). Artemis: sequence visualization and annotation. Bioinformatics.

[B23] Serganov A, Nudler E (2013). A decade of riboswitches. Cell.

